# Designing magnetic field sensor based on tapered photonic crystal fibre assisted by a ferrofluid

**DOI:** 10.1038/s41598-021-93568-z

**Published:** 2021-07-12

**Authors:** Mostafa Taghizadeh, Forough Bozorgzadeh, Marjan Ghorbani

**Affiliations:** 1 Atomic and Molecular Group, Faculty of Physics, Yazd University, Yazd, Iran; 2 Physics Department, College of Sciences, Shiraz University, Shiraz, Iran; 3 Faculty of Physics, University of Tabriz, Tabriz, Iran; 4 Stem Cell Research Center, Tabriz University of Medical Sciences, Tabriz, Iran

**Keywords:** Optical materials and structures, Optical physics, Optics and photonics, Optofluidics, Nanoscale materials

## Abstract

A novel magnetic field sensor is proposed based on the combination of in-line tapered photonic crystal fibre (PCF) Mach–Zehnder interferometer and magnetic nanoparticles. The sensor is theoretically investigated and experimentally realized. The effect of the mechanical strain and the magnetic field on the sensitivity of the sensor is studied. It is found that the proposed sensor shows a wavelength-sensitivity of $-\,0.072\,\text {nm/mT}$ and a strain-sensitivity of $1\,\text {pm/}\upmu \,\epsilon \,$. To evaluate the effect of the magnetic nanoparticles on the output light intensity, the sensitivity response of the device has been measured under different magnetic field strengths for different length scales. The experimental results show refractive index changes of the magnetic nanoparticles-infiltrated PCF—acting as fibre cladding—under the applied magnetic field leads to variations of the interferometric output. The sensitivity of magnetic field measurement with the sensor with $30\,\text {mm}$ and $40\,\text {mm}$ PCF could reach up to $0.021\,\text {dB/mT}$ and $0.017\,\text {dB/mT}$, respectively. The results show a very good linear response that is an essential requirement for the practical sensors. The proposed magnetic field sensor finds applications in various areas, such as optical sensing, military, power industry, and tunable photonic devices.

## Introduction

With the advent of nanotechnology, a new class of smart nano-materials emerges as a promising platform for novel optoelectronic devices. In particular, ferrofluids (FFs) or magnetic fluids, which are stable colloidal solutions containing surfactant-coated magnetic nanoparticles dispersed in an appropriate liquid, possess both the magnetism of magnetic nanoparticles and fluidity of liquid materials^1,2^. The unique properties of FFs, such as optical anisotropy and birefringence, the Faraday effect, tunable refractive index, and field-dependent transmission, can be tuned by external magnetic fields. Due to these outstanding magneto-optical properties, FFs have been used to design unique photonic devices, such as optical switches^3^, optical modulators^4^, tunable optical filters^5^, optical fibre gratings^6^, and sensors^7,8^.

Photonic crystal fibres (PCFs), first invented and demonstrated in 1996^9^, have peculiar properties owing to the complex pattern of air-holes in their cross-section that runs within the fibre. The intrinsic air-holes of the PCF cladding that can be infiltrated by gases, dyes, liquids, or low-viscosity polymers^10,11^, establish an adaptable platform for developing tunable optofluidic devices. For example, a tunable electro-optical modulator has been realized by filling one air-hole of PCF with liquid-crystal^12^. Also, it is shown that the infiltration of optofluids into the first ring of the PCF cladding may lead to dispersion engineering for tunable wavelength conversion^13^. The temperature and refractive index sensors based on in-line Fabry–Pérot interferometer in PCF were reported^14^. It is shown that the sensitivity can be increased via optimizing the length of PCF. Tapered PCF was employed in an interferometer structure to construct a miniaturized refractive index sensor^15^. Using the advantages of polarization-maintaining PCFs, a magnetic field sensor with the sensitivity of $242\,\text { pm/mT}$ is reported^16^.

Accurate measurements of the magnetic field are of scientific and industrial importance for many applications such as medicine, military, vehicle monitoring, and geological prospecting. In the last few decades, various types of magnetic field sensor are proposed and realized, for example, based on side-polished fibre^17^, cascaded microfibre coupler^18^, fibre ring cavity laser^19^, tapered microfibre^20^, and birefringence in liquid-core optical waveguide^21^. Recently, the plasma resonance effect is used in a twin-core PCF to realise the simultaneous measurement of magnetic field and temperature^22^. Also, the magnetic field sensitivity and resolution of a surface Plasmon resonance (SPR)-based PCF magnetic field sensor is theoretically analysed while optimizing the sensor structure parameters^23^. An innovative sensor based on tapered few-mode fibre (TFMF) and Magnetic fluid encapsulated outside the TFMF is proposed to detect magnetic fields in different directions^24^. The sensitivity response of the magnetic field sensors had reported in terms of changes in wavelength shift or output intensity. Among different kinds of magnetic field sensors, fibre-optic interferometric sensors are the ones that have attracted a great deal of research attention due to the compactness, immunity to electromagnetic interference, low-cost and easy fabrication. A fibre-optic interferometer operates based on the interference between two propagating beams either in two different adjacent fibres or through different optical paths in a single optical fibre. Especially, the unique properties of PCFs make them appealing for optical sensing. PCF-based interferometric sensors include Fabry–Pérot interferometer, Mach–Zehnder interferometer, Michelson interferometer, and Sagnac interferometer^25–27^. In particular, to implement an in-line Mach–Zehnder interferometer (MZI), a beam splitter, and a beam combiner are needed to guide light in the arms of the interferometer^28^. Generally, an in-line fibre-based interferometer can be realized via core misalignment and collapsing methods. In these methods, a more complicated interference pattern is expected due to the possibility of exciting the high-order cladding modes^29^.

In this work, we report a low-cost highly-sensitive magnetic field sensor using the advantages of an in-line MZI in an FF-assisted tapered PCF. To the best of our knowledge, this is the first time that the magnetic sensitivity of a tapered PCF-MZI is reported. The sensor behavior has been investigated both theoretically and experimentally. In the first step, the $\text {F}{{\text {e}}_{\text {3}}}{{\text {O}}_{\text {4}}}$ magnetic nanoparticles are synthesized using a reverse co-precipitation method. Then, by accurately injecting a small amount of the FF into two innermost air-holes of PCF and exposing the PCF-MZI to the magnetic field, the refractive index variations are verified. Thus, a magnetically-tunable refractive index can lead to changes in output light. The PCF is then tapered using a custom-made tapering machine. It is found that the proposed sensor shows a strain sensitivity of $1\,\text {pm/}\,\upmu \,\,\varepsilon \,$. For a given length of tapered-PCF, it is shown that the sensitivity of the sensor depends on the applied magnetic field. It is found that the sensitivity is strongly dependent on the length of the MZI. We have shown that the magnetic field of few mT can be detected with higher sensitivity of $0.024\,\text {dB/mT}$. Compared with other magnetic field sensors, the proposed PCF-MZI sensor has many features, such as a simple compact structure, high sensitivity, and stability. Moreover, since splicing a section of a PCF in between two common single-mode fibres (SMFs) is not complex, the fabrication is simple and cost-effective, making it a good candidate for multi-parameter measurements. The results show a very good linear response that is an essential requirement for the practical sensors.

## Materials and methods

### Synthesis of $\text {F}{{\text {e}}_{\text {3}}}{{\text {O}}_{\text {4}}}$ magnetic nanoparticle

Iron oxide ($\text {F}{{\text {e}}_{\text {3}}}{{\text {O}}_{\text {4}}}$) magnetic nanoparticles of average size $\sim$10–12 nm were synthesized using the reverse co-precipitation method^16^, which is an efficient low-cost method. Note that in the normal co-precipitation method, the precipitating agent is added to the cation solution that causes the pH value to increase from acidic state to basic state, whereas in the reverse co-precipitation this addition is inversed^30^. All chemicals used in the experiment were purchased from Sigma-Aldrich. In the synthesis, the base solution is prepared by diluting 30% ammonia ($\text {N}{{\text {H}}_{\text {4}}}\text {OH}$) with adding doubled distilled water (DDW) (3:2 ratio) in a three-neck flask. The solution is mechanically stirred and heated to $80\,^{\circ }\text {C}$ under argon. Then, Fe salt solution is prepared by dissolving ferrous sulfate heptahydrate ($\text {FeS}{{\text {O}}_{\text {4}}}\cdot \text {7}{{\text {H}}_{\text {2}}}\text {O}$) and Iron(III) nitrate $\text {Fe(N}{{\text {O}}_{\text {3}}}{{\text {)}}_{\text {3}}}$ (with 1:2 molar ratio) in $200\,\text {ml}$ DDW. While keeping the temperature constant at $80\,^{\circ }\text {C}$, these solutions were mixed under vigorous stirring for 30 min under argon. The final solution was turned from brown to black indicating the nucleation and growth of magnetic nanoparticles. After rapidly cooled down to room temperature using an ice bath, the solution is carefully injected inside the PCF air-holes using a syringe with a 0.5 mm diameter needle. The FF-infiltrated PCF has been left for $24\,\text {h}$ before any further steps.

### Fabrication and sensing principle

**Figure 1:**
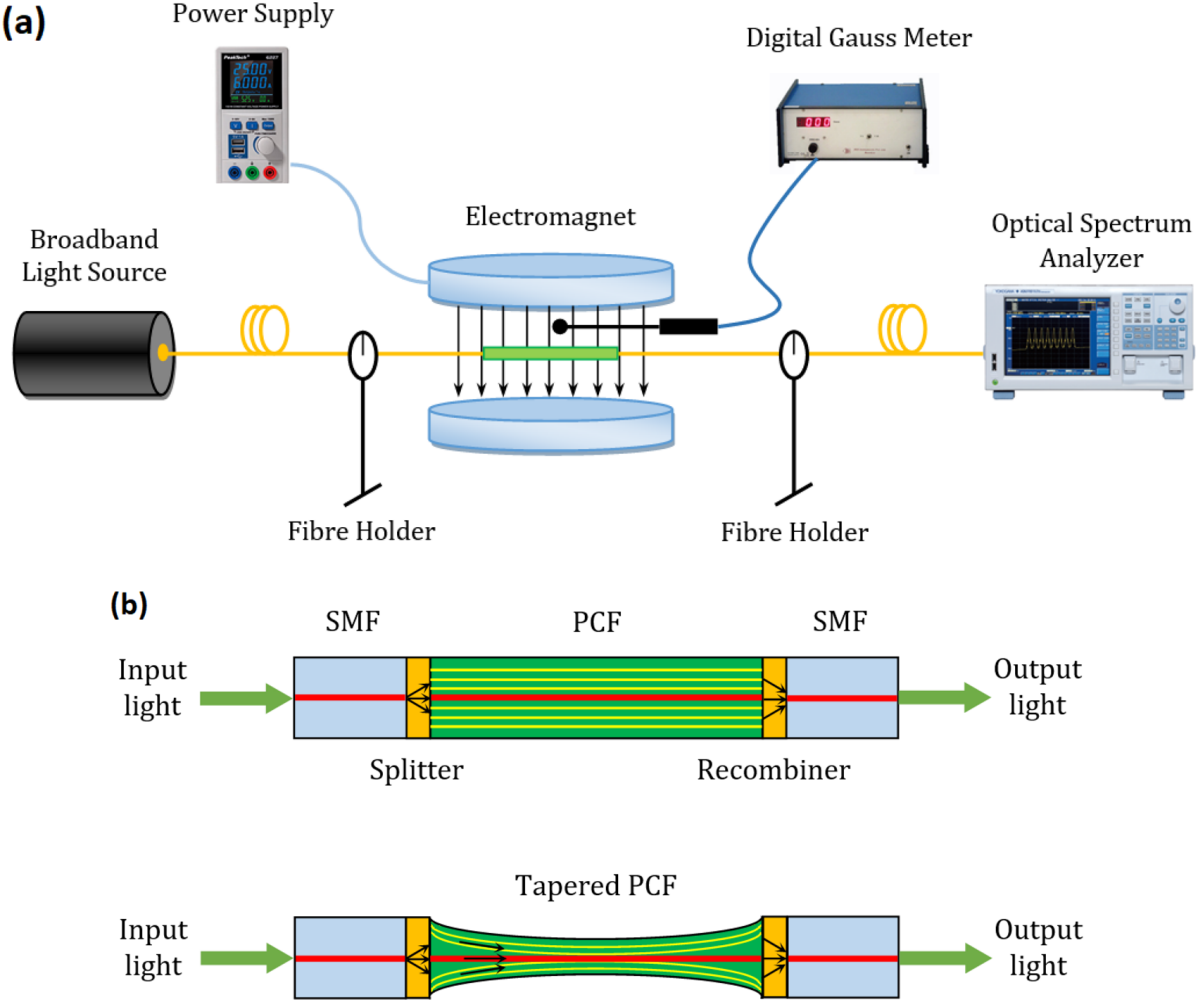
Schematic diagram of (**a**) the experimental setup for magnetic field sensing, (**b**) the in-line Mach–Zehnder interferometer in tapered PCF.

The experimental setup of the magnetic field sensor is shown in Fig. 1a. The sensor structure consists of a broadband light source (BLS) (Thorlabs, SLS202(/M)), a sensing PCF-based interferometer, an optical spectrum analyzer (OSA) (Yokogawa, AQ6370D). As shown in Fig. 1b, to implement the in-line PCF-MZI, a small section of PCF is spliced between a lead-in and a lead-out standard SMF-28 fibre. The PCF used in this experiment is a large-mode-area PCF (Thorlabs, LMA-35) which has a core diameter of $35\, \upmu \text { m}$, outer cladding diameter of $335\,\upmu \text { m}$, $\Lambda =23.15\,\,\upmu \text { m}$, $d/\Lambda =0.5$ and the attenuation of $<0.01\,\text {dB/m}$ @$1550\,\text {nm}$. The fusion splicing is performed with a commercial fusion splicing machine (Ericsson FSU-955). The length of the collapsed zones is carefully controlled by changing the intensity and the duration of arc discharge. In the fabricated PCF-MZI, one propagating beam is referred to as the sensing arm and the other one is called the reference arm^26^. Even though the physical length of the two arms is identical, the interference signal can be detected. Physically, this behavior can be understood from the fact that since the effective indices of the core and cladding are not identical, there will be an optical path difference between the arms of the interferometer. In this case, the phase velocity of the reference and sensing arm is different.

Since the MZI-based sensors are very sensitive to fibre bending, the device was firmly kept constant with the aid of fibre holders. To apply the axial strain, the two sides of inline PCF-MZI were clamped to two stages: a fibre holder of the micro-positioner stage and a fibre holder of the fixed stage. The PCF-MZI is then placed between two poles of an electromagnet, which generates a uniform magnetic field. The intensity of the magnetic field can be adjusted by tuning the power supply. The magnetic field direction is perpendicular to the propagation of light and the optical fibre axis. The operating principle of the sensor is as follows: Light from the BLS is coupled into the lead-in SMF and enters the collapsed zone of the PCF. In this region, light promptly begins to diffract, leading to mode broadening. Then it passes through the PCF region, recombines at the second collapsed zone. The output light is monitored and analyzed by the OSA with the wavelength resolution 0.02 nm. The occurrence of total internal reflection and more importantly the interference between core and cladding modes is dependent on the axial strain, the strength of the magnetic field, and the length of the MZI.

The device can be modeled using a core and cladding mode interference equation given by^29^

1 $$\begin{aligned} {{I}_{\text {out}}}={{I}_{\text {1}}}+{{I}_{\text {2}}}+2\sqrt{{{I}_{\text {1}}}{{I}_{\text {2}}}}\cos \left( \Delta \Phi \right) , \end{aligned}$$

where ${{I}_{\text {out}}}$ is the intensity of the total interference signal, ${{I}_{\text {1}}}$ and ${{I}_{\text {2}}}$ are the intensities of light propagating in the fibre core and cladding. The phase difference between the core and cladding modes, $\Delta \Phi$, depends on the effective refractive index difference and the physical length of the interferometer, and can be expressed as

2 $$\begin{aligned} \Delta \varphi =\frac{2\pi \,}{\lambda }\Delta \,{{n}_{\text {eff}}}(\lambda )\,L, \end{aligned}$$

where $\Delta {{n}_{\text {eff}}}=n_{\text {eff}}^{\text {core}}-n_{\text {eff}}^{\text {clad}}$ is the effective refractive index difference between the core and cladding, *L* is the length of the interferometer, and $\lambda$ is the central wavelength of the BLS. As a result of the magnetic field dependence of the FF-filled cladding refractive index, this phase difference also depends on the applied magnetic field.

The minimum in the intensity of the output spectrum will occur when

3 $$\begin{aligned} {{\lambda }_{\min }}=\frac{2\,\Delta {{n}_{\text {eff}}}L}{2\text {m}+1}, \end{aligned}$$

where $\text {m}$ is the interference order. In the absence of an external magnetic field, the coupling length, $\zeta$, can be obtained by using the propagation constants of fundamental and cladding modes ${{\beta }_{\text {core}}}$ and ${{\beta }_{\text {clad}}}$ as $\zeta =2\pi /({{\beta }_{\text {core}}}-{{\beta }_{\text {clad}}})=\lambda /({{n}_{\text {core}}}-{{n}_{\text {clad}}})$^31^. The low difference between refractive indices is responsible for the high coupling length and also responsible for high sensitivity^32^.

## Experimental results and discussion

**Figure 2:**
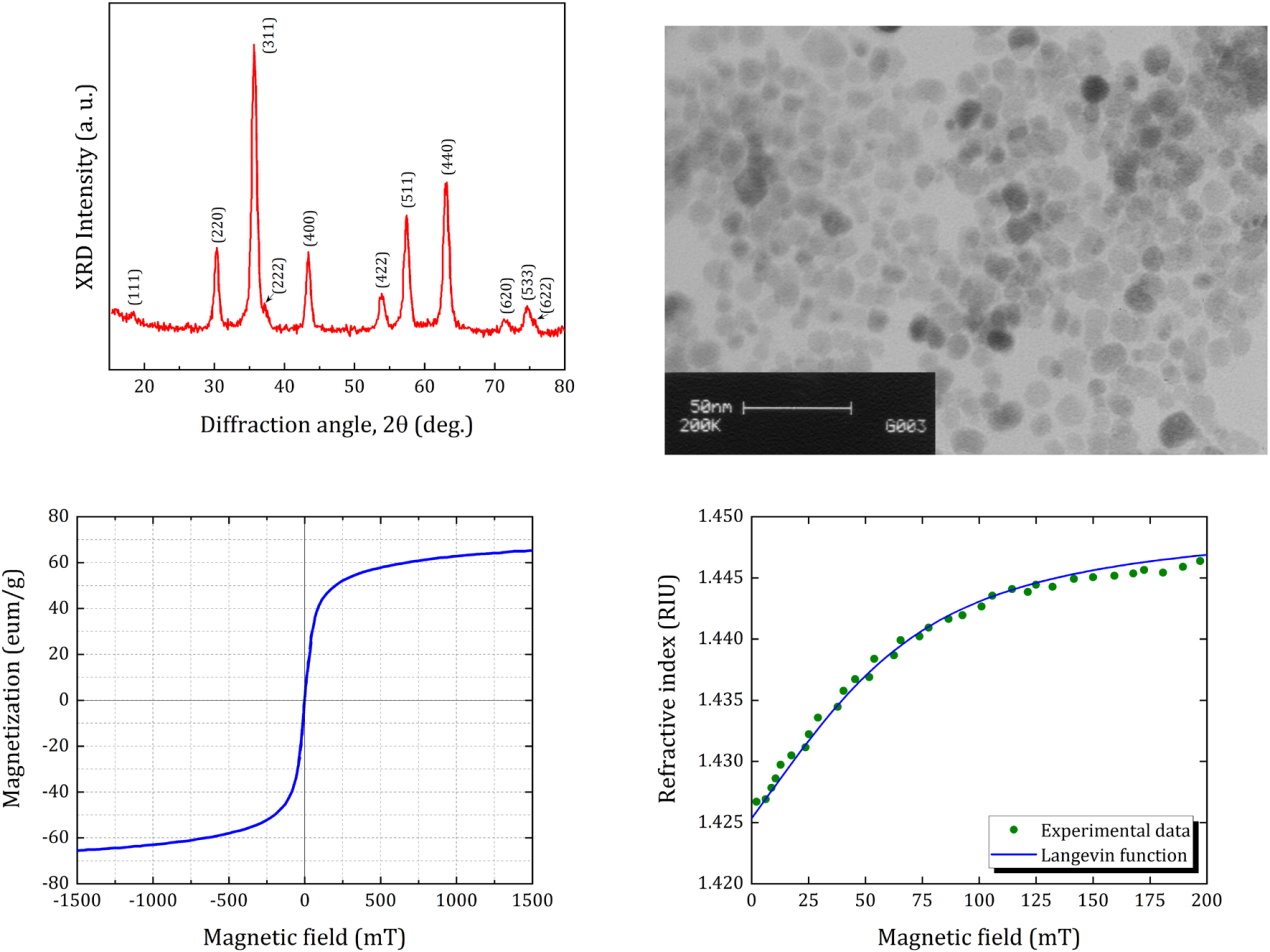
(**a**) X-ray diffraction pattern and (**b**) TEM image for as-synthesized $\text {F}{{\text {e}}_{\text {3}}}{{\text {O}}_{\text {4}}}$ magnetic nanoparticles. (**c**) Room-temperature magnetization curve and (**d**) Refractive index of ferrofluid versus magnetic field intensity.

In this section, the main results are presented and the effect of magnetic field and strain on the intensity of output light is investigated. The FF used in our study is $\text {F}{{\text {e}}_{\text {3}}}{{\text {O}}_{\text {4}}}$ nanoparticles synthesized by the chemical reverse co-precipitation method. The crystalline nature of the synthesized magnetic nanoparticles is characterized by X-ray diffraction (XRD) analysis, shown in Fig. 2a. The observed diffraction peaks at $2\theta =\,{{30.35}^{\circ }},\,{{35.63}^{\circ }},\,{{37.07}^{\circ }}\text {,}\,{{43.40}^{\circ }},\,{{53.87}^{\circ }},\,{{57.41}^{\circ }}$ and ${{63.17}^{\circ }}$ corresponds to (hkl) = (220), (311), (222), (400), (422), (511), and (440) planes of $\text {F}{{\text {e}}_{\text {3}}}{{\text {O}}_{\text {4}}}$ in cubic phase, which is in a good agreement with the literature Joint Committee on. Powder Diffraction Standards (JCPDS card, No. 19-0629). The average size of crystallite domains *D* calculated using the Debye-Scherrer equation $D=K\lambda /(\beta \cos \theta )$ is 11.56 nm that confirmed by transmission electron microscopy (TEM). Here, $K=0.9$ is a dimensionless shape factor, $\lambda =0.15406\,\text {nm}$ is the X-ray wavelength, $\beta$ is the line broadening at half the maximum intensity (FWHM), and $\theta$ is the Bragg angle. Figure 2b shows a TEM image of synthesized $\text {F}{{\text {e}}_{\text {3}}}{{\text {O}}_{\text {4}}}$ in which most of the nanoparticles are spherical with an average diameter of $\sim$10–12 nm.

Figure 2c shows the magnetization curve for as-synthesized $\text {F}{{\text {e}}_{\text {3}}}{{\text {O}}_{\text {4}}}$ magnetic nanoparticles. The saturation magnetization measured at $300\,^{\circ }\text {K}$ is about $65\,\text {emu/g}$, exhibiting conformity with previous works^33,34^. It is known that the optical response of the FFs depends on temperature and magnetic field intensity and simultaneous changes in these parameters cause an inaccurate magnetic field sensing^35^. The relationship between refractive index and magnetic field for $\text {F}{{\text {e}}_{\text {3}}}{{\text {O}}_{\text {4}}}$ FF has been extensively reported in the literature^36,37^, and it is found that the refractive index of the magnetic fluid increases with enhancing field strength^38^. Figure 2d shows the magnetic field dependence of the FF refractive index ${{n}_{FF}}$. The refractive index is measured via the direct contact angle method^39^. It is observed that ${{n}_{FF}}$ is constant until the applied magnetic field reaches a critical value ${{H}_{c}}=8\,\text {mT}$. Beyond ${{H}_{c}}$, the ${{n}_{FF}}$ increases until it reaches saturation. Therefore, the operating range of the sensor is in the range of $8\,\text {mT}$ to $140\,\text {mT}$. Under a constant temperature *T*, ${{n}_{FF}}$ can be obtained from the Langevin function^40,41^.

4 $$\begin{aligned} {{n}_{FF}}\left( H,T \right) =\left( {{n}_{f}}-{{n}_{i}} \right) \left[ \coth \left( \alpha \frac{H-{{H}_{c}}}{T} \right) -\frac{T}{\alpha \left( H-{{H}_{c}} \right) } \right] +{{n}_{i}},\,\,\,\,\,\,\,\,\,\,\,\text {for}\,\,H>{{H}_{c}}, \end{aligned}$$

where ${{n}_{i}}$(= 1.4254 here) is the refractive index of the FFs under magnetic fields lower than ${{H}_{c}}$, ${{n}_{f}}$(= 1.4469) is the saturated value of the FF refractive index, and $\alpha$ is a fitting coefficient. Note that ${{H}_{c}}$ depends on the concentration of FFs and the type of carrier liquid^40^.

**Figure 3:**
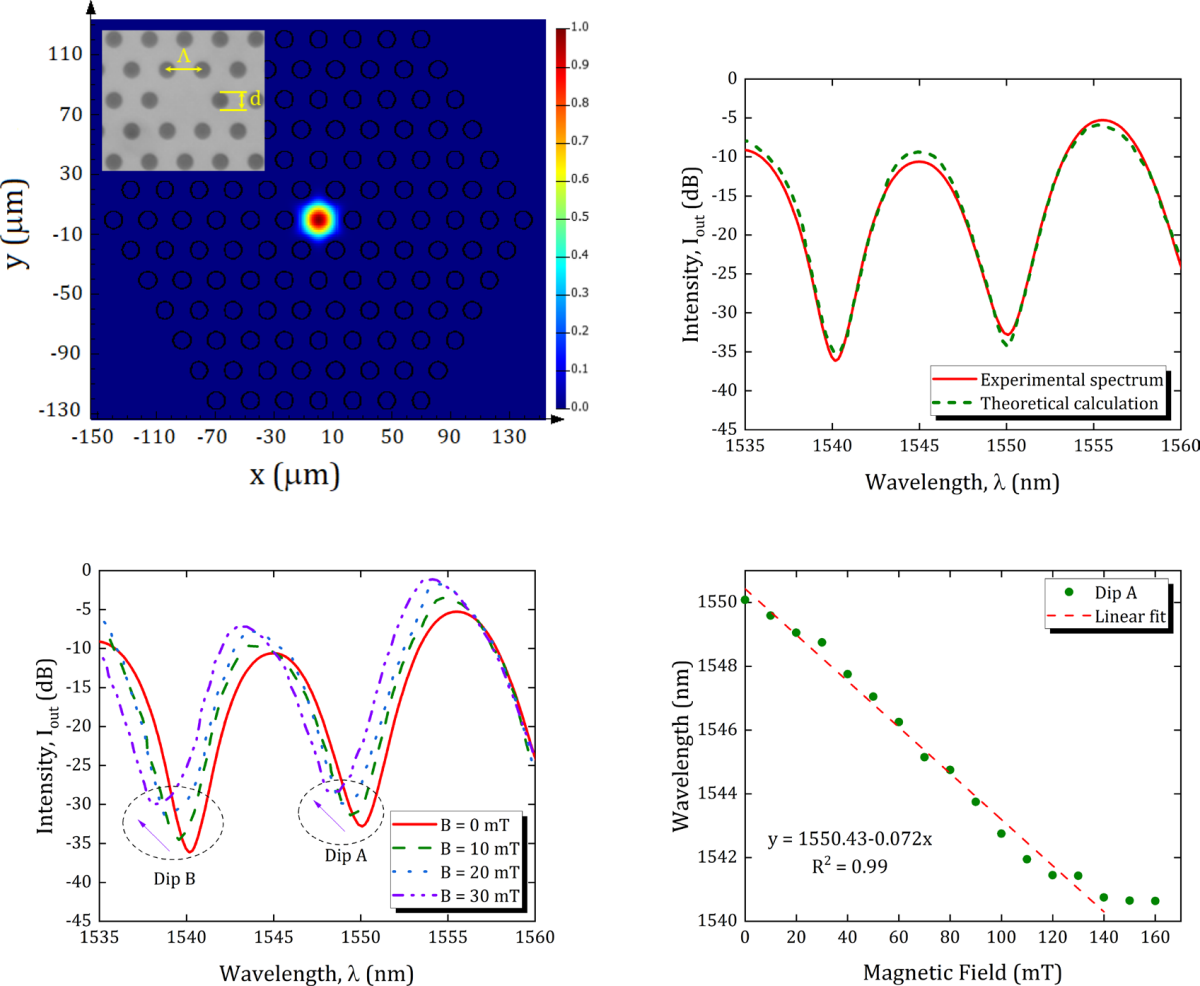
(**a**) Calculated field distributions of PCF core mode at 1550 nm wavelength, (**b**) comparison between the experimental and theoretical spectrum, (**c**) the output intensity as a function of wavelength for different magnetic fields, and (**d**) wavelength shift of Dip A versus magnetic-field intensity.

Prior to the experimental studies, the numerical simulation carried out with the PCF structural parameters as radius of holes of $5.8\,\upmu \text { m}$, the lattice constant of $23.15\,\upmu \text { m}$, the core diameter of $35\,\upmu \text { m}$, and the outer cladding diameter of $335\,\upmu \text { m}$. The mode field diameter of LMA-35 PCF is $26.0\pm 2.5\,\,\upmu \text { m}$, which is almost three times that of a SMF. Figure 3a shows the fundamental core mode of the PCF. The simulation is carried out using commercial software (ANSYS Lumerical 2020). The transmission spectrum of the PCF-MZI in the absence of an external magnetic field is presented in Fig. 3b. It can be observed that the results obtained from the experiment show a very good agreement with the theoretical result. The interferometry spectrum of the PCF-MZI with increasing magnetic field from 0 to $30\,\text {mT}$ is experimentally obtained and presented in Fig. 3c. During the experiment, both ends of the magnetic field sensor were held straight to prevent the effect of strain variations. It can be realized that the intensity dips are narrow and interference dips shift towards short wavelengths (i.e. dips experience blue-shift), which is consistent with the reduction of $\Delta {{n}_{\text {eff}}}$ by increasing magnetic field [see Eq. (3)]. Sensitivity of the sensor can be calculated by $S={\Delta \lambda }/{\Delta H}\;$ (nm/mT), where $\Delta \lambda$ is the shift of dip wavelength and $\Delta H$ is the variation of magnetic field intensity. As shown in Fig. 3d, when the magnetic field intensity is less than $140\,\text {mT}$, the sensitivity behavior of the sensor is primarily a linear relationship. In the linear regime, the sensitivity of the magnetic field sensor is found to be $-0.072\,\text {nm/mT}$. Beyond $140\,\text {mT}$, the wavelength shift tends to be constant, as the refractive index of FF gets saturated.

**Figure 4:**
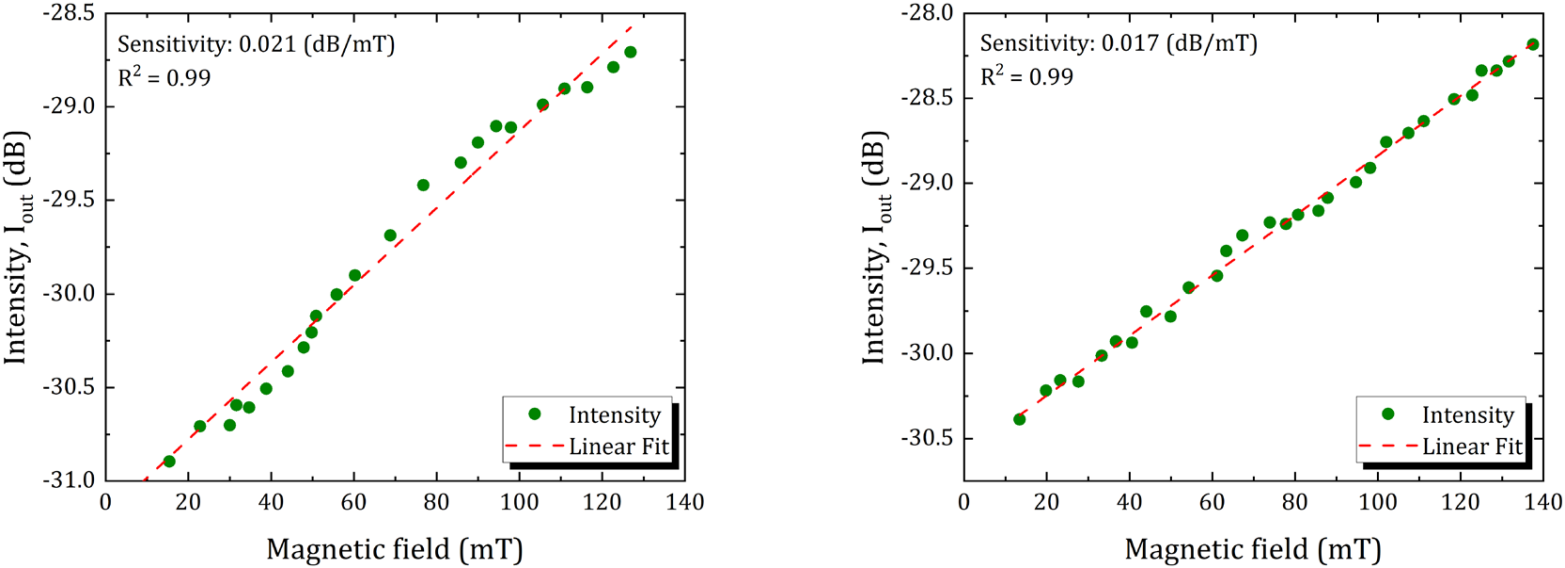
Measurements of the output intensity as a function of magnetic field variation for (**a**) $L=30\,\text {mm}$, and (**b**) $L=40\,\text {mm}$.

When the involved modes propagate in the PCF, the effective refractive index difference between the core mode and corresponding cladding mode results in an optical phase difference (OPD). Due to the OPD in the sensing and reference arm, interference occurs between the light beams in these arms. Filling the FF solution into the air hole of the PCF reduces the effective refractive index difference between the cladding and the core of the PCF, thereby increasing the sensitivity of sensing structure to the external environment^42^. As mentioned before, the proposed sensor operates based on light intensity modulation. It is well-known that the refractive index of PCF background material (i.e. Corning 7980 fused Silica) is not sensitive to the magnetic field. However, the randomly homogenous structure of FFs changes under an external magnetic field. Therefore, field-dependent rearrangement of $\text {F}{{\text {e}}_{\text {3}}}{{\text {O}}_{\text {4}}}$ nanoparticles leads to the refractive index variation of the FF, accordingly, light modulation is possible. The intensity dips can also be shifted by changing the length of PCF (*L*). In order to study the dependence of the Dip A intensity on the physical length of PCF-MZI, two interferometers with different PCF lengths were fabricated. The effect of magnetic field strength on the output light intensity is investigated by gradually increasing the applied magnetic field from 0 to $140 \,\text {mT}$. Figure 4 shows the variation of intensity dip as a function of magnetic field strength. The magnetic field dependence of the intensity dip is obtained for two different lengths of PCF-MZI. It is found that the dip intensity of output light is an ascending function of the applied magnetic field. The experimental results show that the sensitivity of magnetic field measurement could reach $0.021\,\text {dB/mT}$, and $0.017\,\text {dB/mT}$ for the PCF-MZI lengths of $L=30\,\text {mm}$ and $L=40\,\text {mm}$, respectively. From Fig. 4a, it can be predicted that the intensity dip of output light changes slightly for the magnetic field below $8\,\text {mT}$, which is in agreement with previous results^43^. It is worth mentioning that the intensity of the output light tends to be constant for the magnetic strength range beyond the saturation magnetization of $\text {F}{{\text {e}}_{\text {3}}}{{\text {O}}_{\text {4}}}$ magnetic nanoparticles, which is not investigated in this study.

**Figure 5:**
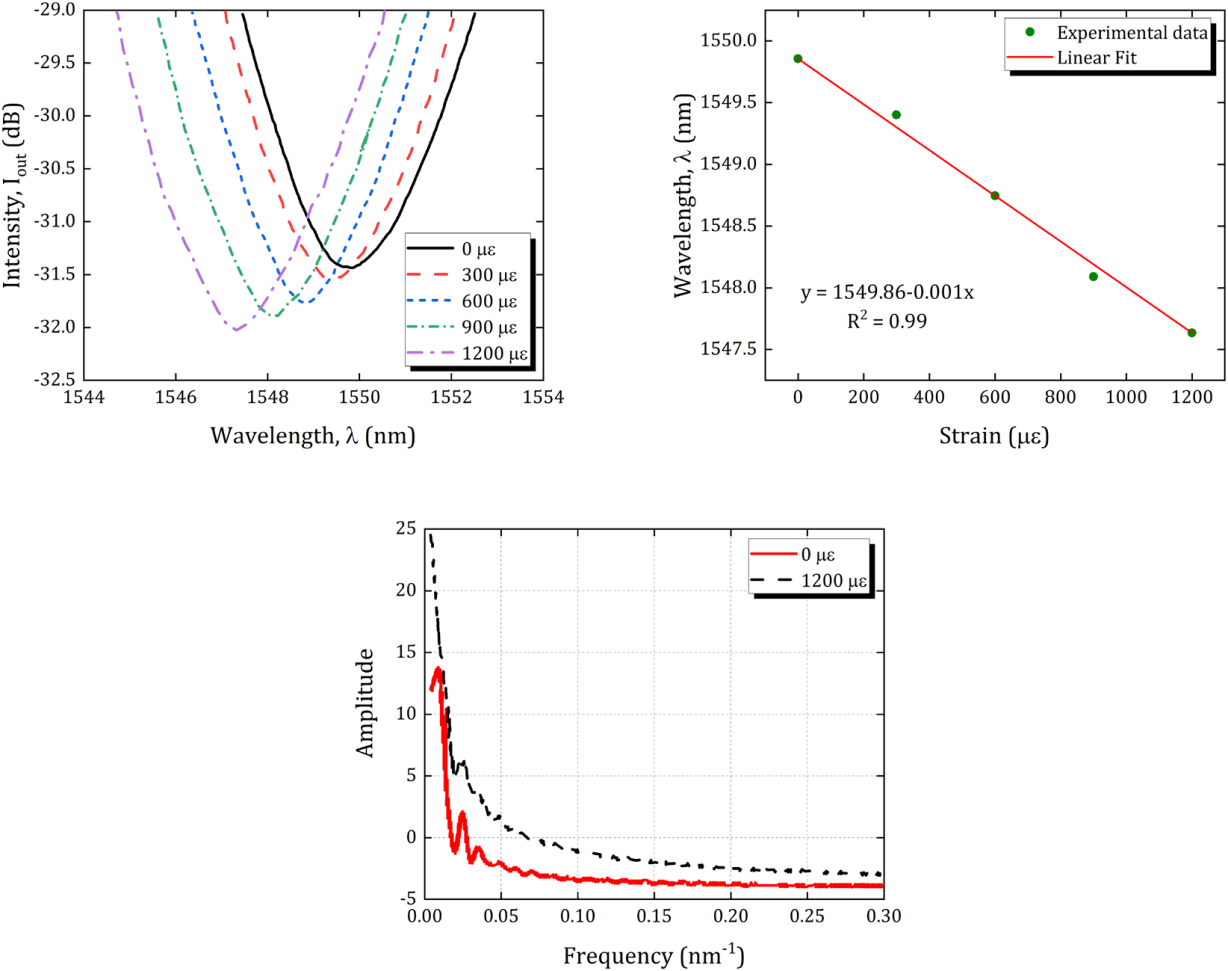
(**a**) The output intensity of PCF-MZI as a function of wavelength, (**b**) The wavelength shift as a function of the axial strain, and (**c**) The effect of axial strain on the spatial frequency spectra.

In the next step, to investigate the wavelength-strain sensitivity of the sensor, the two sides of the inline PCF-MZI were attached to two stages, a moving stage and a fixed one. The axial movement of the micro-positioner stage causes an axial strain on the tapered region along with other PCF section. This leads to a wavelength shift of the interference pattern. Figure 5a shows the axial strain response of the in-line PCF-MZI sensor. From the transmission spectrum of the PCF-MZI, it can be observed that the intensity dip is blue-shifted without a significant change in its shape as the axial strain is increased from 0 to $1200\,\,\upmu \text { }\varepsilon \,$. The changes in fibre dimensions and the photo-elastic effect of the fibre due to the applied axial strain lead to this shift in the interferometric spectrum. At a constant temperature, the wavelength shift due to the strain can be expressed as $\Delta \lambda /\lambda =-\left( 1+2\nu +{{p}_{e}} \right) \varepsilon$, where $\nu$ is the Poisson ratio of the fibre, ${{p}_{e}}$ is the effective strain-optic coefficient, and $\varepsilon =\Delta L/L$ is the axial strain applied on the fibre^44^. The relationship between the applied strain and the dip wavelength is presented in Fig. 5b and the strain sensitivity is found to be $1\,\text { pm/}\upmu \,\varepsilon \,$. A fitting curve of the experimental data shows that the wavelength shift is linear with the correlation factor of ${{\text {R}}^{\text {2}}}=0.99$. To find out the number and power distribution of the interfering modes, the fast Fourier transform (FFT) of transmission spectrum is taken to obtain its corresponding spatial frequency spectra. Figure 5c shows that only one cladding mode is dominantly excited, while the other cladding modes are very weak. It can be observed that applying the strain on the sensor changes the interferometer length, leading to the increment of the power of the cladding modes.

**Table 1:** Sensitivities of some of the PCF-based magnetic field sensors.

Sensor structures	Sensitivity	References number
MF-enhanced Dual-S-Shaped structure	0.2904 nm/mT	^45^
MF-infiltrated dual-core PCF	4.80 pm/Oe	^46^
Selectively FF-infiltrated dual-core PCF	305.8 pm/Oe	^47^
Single-mode (SM)- multimode (MM) -PCF-SM MZI sensor	$-$0.13 dB/mT	^48^
SM–MM–SM fibre structure	$-$16.86 pm/Oe	^49^
PCF-based MZI with FF-filled microhole	0.42 dB/mT	^50^
SM-PCF-SM	$-$0.303 dB/mT	^51^
D-shaped PCF	0.87 nm/mT	^52^
MZI-integrated MM-SM-MM fibre	0.123 nm/mT	^53^
MF-filled PCF	924.63 pm/mT	^42^
Honeycomb PCF	4.3763 dB/Oe	^54^
Tapered PCF-MZI	0.021 dB/mT $-$0.072 nm/mT	This work

Table 1 provides a brief review of the sensitivities of PCF-based magnetic field sensors. Compared to the previous intensity-modulated magnetic field sensors, the proposed sensor has a higher sensitivity for low magnetic field intensity measurement. At the same time, the sensor exhibits linear response that is an essential requisite for the practical sensors.

## Conclusion

In conclusion, we have demonstrated a highly-sensitive magnetic field sensor based on the combination of an in-line PCF-MZI with the characteristics of the magnetic nanoparticles. First, the $\text {F}{{\text {e}}_{\text {3}}}{{\text {O}}_{\text {4}}}$ ferrofluid is synthesized using the reverse co-precipitation method. The FF response to the magnetic field intensity exhibits a Langevin function. By infiltrating air-holes of the PCF with FF and fusion splicing with standard SMFs, in-line PCF-MZI is realized. The effect of the magnetic field and the mechanical strain on the sensitivity of the sensor is studied. It is found that the proposed sensor shows a wavelength-sensitivity of $-0.072\,\text {nm/mT}$ and a strain-sensitivity of $1\,\text {pm/}\upmu \,\varepsilon \,$. The experimental results show refractive index changes of the FF-filled PCF - acting as fibre cladding - under the applied magnetic field leads to variations of the interferometric output. The sensitivity of magnetic field measurement could reach up to $0.017 \,\text {dB/mT}$. The results show a very good linear response that is an essential requirement for the practical sensors. The proposed magnetic field sensor finds applications in various areas, such as optical sensing, military, power industry, and tunable photonic devices.

## References

[1] Gubin SP, Koksharov YA, Khomutov GB, Yurkov GY (2005). Magnetic nanoparticles: preparation, structure and properties. Russian Chemical Reviews.

[2] Luo L, Pu S, Dong S, Tang J (2015). Fiber-optic magnetic field sensor using magnetic fluid as the cladding. Sensors and Actuators A: Physical.

[3] Horng HE (2004). Tunable optical switch using magnetic fluids. Applied Physics Letters.

[4] Horng HE (2005). Designing optical-fiber modulators by using magnetic fluids. Opt. Lett..

[5] Liu T (2007). Tunable magneto-optical wavelength filter of long-period fiber grating with magnetic fluids. Applied Physics Letters.

[6] Candiani A, Margulis W, Sterner C, Konstantaki M, Pissadakis S (2011). Phase-shifted bragg microstructured optical fiber gratings utilizing infiltrated ferrofluids. Opt. Lett..

[7] Zhao Y, Liu X, Lv R-Q, Zhang Y-N, Wang Q (2017). Review on optical fiber sensors based on the refractive index tunability of ferrofluid. J. Lightwave Technol..

[8] Cennamo N (2021). A magnetic field sensor based on spr-pof platforms and ferrofluids. IEEE Transactions on Instrumentation and Measurement.

[9] Knight JC, Birks TA, Russell PSJ, Atkin DM (1996). All-silica single-mode optical fiber with photonic crystal cladding. Opt. Lett..

[10] Martelli C (2009). Evanescent-field spectroscopy using structured optical fibers: Detection of charge-transfer at the porphyrin-silica interface. Journal of the American Chemical Society.

[11] Markos C, Vlachos K, Kakarantzas G (2010). Bending loss and thermo-optic effect of a hybrid pdms/silica photonic crystal fiber. Opt. Express.

[12] Huang Y (2019). Tunable electro-optical modulator based on a photonic crystal fiber selectively filled with liquid crystal. Journal of Lightwave Technology.

[13] Pakarzadeh H, Derakhshan R, Hosseinabadi S (2019). Tunable wavelength conversion based on optofluidic infiltrated photonic crystal fibers. Journal of Nonlinear Optical Physics & Materials.

[14] Dash JN, Jha R (2015). Inline microcavity-based pcf interferometer for refractive index and temperature sensing. IEEE Photonics Technology Letters.

[15] Ahmed F, Ahsani V, Melo L, Wild P, Jun MBG (2016). Miniaturized tapered photonic crystal fiber Mach–Zehnder interferometer for enhanced refractive index sensing. IEEE Sensors Journal.

[16] Thakur HV, Nalawade SM, Gupta S, Kitture R, Kale SN (2011). Photonic crystal fiber injected with fe3o4 nanofluid for magnetic field detection. Applied Physics Letters.

[17] Li Y (2019). All-fiber-optic vector magnetic field sensor based on side-polished fiber and magnetic fluid. Opt. Express.

[18] Mao L (2016). Magnetic field sensor based on cascaded microfiber coupler with magnetic fluid. Journal of Applied Physics.

[19] Bai X (2016). Magnetic field sensor using fiber ring cavity laser based on magnetic fluid. IEEE Photonics Technology Letters.

[20] Ma Z (2018). A highly sensitive magnetic field sensor based on a tapered microfiber. IEEE Photonics Journal.

[21] Wang W, Zhang H, Li B, Li Z, Miao Y (2019). Optical fiber magnetic field sensor based on birefringence in liquid core optical waveguide. Optical Fiber Technology.

[22] Liu H, Chen C, Wang H, Zhang W (2020). Simultaneous measurement of magnetic field and temperature based on surface plasmon resonance in twin-core photonic crystal fiber. Optik.

[23] Huang, H. *et al.* A highly magnetic field sensitive photonic crystal fiber based on surface plasmon resonance. *Sensors* **20**, 10.3390/s20185193 (2020). 10.3390/s20185193 PMC7570832 32933069

[24] Fu X (2021). A multi-directional magnetic field sensor based on tapered few mode fiber and magnetic fluid. Optik.

[25] Ding W, Jiang Y, Gao R, Wang Z, Liu Y (2014). An in-line photonic crystal fibre-based mach–zehnder interferometer with temperature compensation. Measurement Science and Technology.

[26] Hu, D. J. J., Wong, R.Y.-N. & Shum, P. P. Photonic Crystal Fiber-Based Interferometric Sensors. *Select. Top. Opt. Fiber Technol. Appl.*10.5772/intechopen.70713 (2018). (InTech)

[27] Shao M, Sun H, Liang J, Han L, Feng D (2021). In-fiber michelson interferometer in photonic crystal fiber for humidity measurement. IEEE Sensors Journal.

[28] Wei, T., Lan, X., Zhang, Y. & Xiao, H. Fiber inline core-cladding-mode interferometer fabricated by CO2 laser irradiation. In Xiao, H., Fan, X. & Wang, A. (eds.) *Photonic microdevices/microstructures for sensing*, vol. 7322, 7 – 11, 10.1117/12.818624. International Society for Optics and Photonics (SPIE, 2009).

[29] Choi HY, Kim MJ, Lee BH (2007). All-fiber Mach–Zehnder type interferometers formed in photonic crystal fiber. Opt. Express.

[30] Sangian H, Mirzaee O, Tajally M (2017). Reverse Chemical Co-Precipitation: An Effective Method for Synthesis of BiFeO3 Nanoparticles. Acerp.

[31] Mortensen NA, Nielsen MD, Folkenberg JR, Petersson A, Simonsen HR (2003). Improved large-mode-area endlessly single-mode photonic crystal fibers. Opt. Lett..

[32] Mitu SA (2020). Design of Magnetic Fluid Sensor Using Elliptically Hole Assisted Photonic Crystal Fiber (PCF). Journal of Superconductivity and Novel Magnetism.

[33] Nemala H (2014). Investigation of magnetic properties of fe3o4 nanoparticles using temperature dependent magnetic hyperthermia in ferrofluids. Journal of Applied Physics.

[34] Ghorbani M, Hamishehkar H, Arsalani N, Entezami AA (2015). Preparation of thermo and pH-responsive polymer@Au/Fe3O4 core/shell nanoparticles as a carrier for delivery of anticancer agent. Journal of Nanoparticle Research.

[35] Xia, J., Wang, F., Luo, H., Wang, Q. & Xiong, S. A magnetic field sensor based on a magnetic fluid-filled fp-fbg structure. *Sensors* **16**, 10.3390/s16050620 (2016). 10.3390/s16050620 PMC4883311 27136564

[36] Yang SY (2002). Magnetically-modulated refractive index of magnetic fluid films. Applied Physics Letters.

[37] Horng HE, Hong C-Y, Yang SY, Yang HC (2003). Designing the refractive indices by using magnetic fluids. Applied Physics Letters.

[38] Chen LX, Huang XG, Zhu JH, Li GC, Lan S (2011). Fiber magnetic-field sensor based on nanoparticle magnetic fluid and fresnel reflection. Opt. Lett..

[39] Yoshida K, Ohkubo K, Ojima N, Iwata K (2013). Application of the critical angle method to refractive index measurement of human skin in vivo under partial contact. Journal of Biomedical Optics.

[40] Chen YF (2003). Thermal effect on the field-dependent refractive index of the magnetic fluid film. Applied Physics Letters.

[41] Hong, C.-Y., Horng, H. E. & Yang, S. Y. Tunable refractive index of magnetic fluids and its applications. *Phys. Status Solidi (c)* **1**, 1604–1609, 10.1002/pssc.200304388 (2004). https://onlinelibrary.wiley.com/doi/pdf/10.1002/pssc.200304388.

[42] Wang J (2020). Magnetic field and temperature dual-parameter sensor based on magnetic fluid materials filled photonic crystal fiber. Opt. Express.

[43] Miao Y (2013). Magnetic field tunability of optical microfiber taper integrated with ferrofluid. Opt. Express.

[44] Li E (2007). Temperature compensation of multimode-interference-based fiber devices. Opt. Lett..

[45] Lei XQ, Peng BJ, Chen DR, Shi QG, Ma XW (2016). An all-fiber magnetic field sensor based on dual-s-shaped optic fiber integrated with magnetic fluid. IEEE Sensors Journal.

[46] Li J (2014). Novel magnetic field sensor based on magnetic fluids infiltrated dual-core photonic crystal fibers. Optical Fiber Technology.

[47] Gangwar RK, Bhardwaj V, Singh VK (2016). Magnetic field sensor based on selectively magnetic fluid infiltrated dual-core photonic crystal fiber. Optical Engineering.

[48] Ding XZ (2018). Mach–zehnder interferometric magnetic field sensor based on a photonic crystal fiber and magnetic fluid. Appl. Opt..

[49] Wang H, Pu S, Wang N, Dong S, Huang J (2013). Magnetic field sensing based on singlemode–multimode–singlemode fiber structures using magnetic fluids as cladding. Opt. Lett..

[50] Gao R, Jiang Y (2013). Magnetic fluid-filled microhole in the collapsed region of a photonic crystal fiber for the measurement of a magnetic field. Opt. Lett..

[51] Chen Y, Han Q, Liu T, Yan W, Yao Y (2016). Magnetic field sensor based on ferrofluid and photonic crystal fiber with offset fusion splicing. IEEE Photonics Technology Letters.

[52] Liu H (2018). Temperature-compensated magnetic field sensor based on surface plasmon resonance and directional resonance coupling in a d-shaped photonic crystal fiber. Optik.

[53] qin Lei X, chao Xu Y, ting Yu Y, jin Peng B (2019). Fiber in-line magnetic field sensor based on Mach–Zehnder interferometer integrated with magnetic fluid. Optoelectronics Letters.

[54] Sharma AK, Popescu V (2021). Magnetic field sensor with truncated honeycomb photonic crystal fiber: analysis under the variations in magnetic fluid composition and temperature for high performance in near infrared. Optical and Quantum Electronics.

